# *In silico* modelling to differentiate the contribution of sugar frequency *versus* total amount in driving biofilm dysbiosis in dental caries

**DOI:** 10.1038/s41598-017-17660-z

**Published:** 2017-12-12

**Authors:** David Head, Deirdre A. Devine, P. D. Marsh

**Affiliations:** 1 School of Computing, University of Leeds, Leeds, LS2 9JT United Kingdom; 2 Division of Oral Biology, School of Dentistry, Wellcome Trust Brenner Building, University of Leeds, St James’s University Hospital, Leeds, LS9 7TF United Kingdom; 3 PHE Porton, Salisbury, SP4 0JG United Kingdom

## Abstract

Dental caries is the most prevalent infection globally and a substantial economic burden in developed countries. Dietary sugars are the main risk factor, and drive increased proportions of acid-producing and acid-tolerating (aciduric) bacterial species within dental biofilms. Recent longitudinal studies have suggested that caries is most strongly correlated with total sugar intake, contrasting with the prevailing view that intake frequency is the primary determinant. To explore this possibility, we employed a computational model for supragingival plaque to systematically sample combinations of sugar frequency and total amount, allowing their independent contributions on the ratio of aciduric (*i.e*. cariogenic) to non-aciduric bacteria to be unambiguously determined. Sugar frequency was found to be irrelevant for either very high or very low daily total amounts as the simulated biofilm was predicted to be always or never cariogenic, respectively. Frequency was a determining factor for intermediate total amounts of sugar, including the estimated average human consumption. An increased risk of caries (*i.e*. high prevalence of aciduric/non-aciduric species) was predicted for high intake frequencies. Thus, both total amount and frequency of sugar intake may combine to influence plaque cariogenicity. These findings could be employed to support public guidance for dietary change, leading to improved oral healthcare.

## Introduction

Dental caries, or tooth decay, is the most common infectious disease worldwide^1^; it lowers the quality of life for children and adults alike, and represents a significant economic burden in high-income countries^2,3^. This largely preventable disease is associated with diets that include a regular intake of refined sugars, resulting in the selection of cariogenic bacteria within the dental plaque biofilm^4,5^. Such bacteria are functionally identified as being both acidogenic (metabolizing dietary sugars to organic acids through glycolysis) and aciduric (capable of maintaining growth and high rates of glycolysis in a low pH environment); examples include the mutans streptococci, *Bifidobacterium* spp., and *Lactobacillus* spp.^6^. Repeated acid challenges deriving from regular sugar intake can drive the levels of such cariogenic bacteria from low levels that are clinically insignificant, towards dominance, with a concomitant lowering of pH that initiates the net dissolution of enamel mineral, which ultimately presents as a caries lesion^7,8^. Disease is thus associated with the transition from a symbiotic to a dysbiotic plaque biofilm composition, driven by interactions with the environment and the host, in what can be likened to an ecological catastrophe^9–11^.

The frequency of sugar intake has been held to be the principal driver in the selection of cariogenic bacteria, as typical concentrations of dietary sugar are thought to saturate microbial glycolysis, essentially resulting in utilisation of equal amounts of sugar per episode, while the remaining sugar is removed un-metabolised by saliva flow or converted to extracellular or intracellular polysaccharides. However, it has recently been proposed that the total amount of sugar is also a contributory factor to the caries burden, potentially providing a stronger causal link than that of frequency^12–14^. Evaluating the relative influence of frequency *versus* amount of dietary sugars will be crucial when forming future public guidance on preventative means to reduce caries, in addition to aiding the identification of targets for interventions to reduce the occurrence of carious lesions^15,16^. However, *in vitro* experiments would require extensive assays to systematically characterise all possible combinations of sugar frequency and concentration, and may be confined to short durations of limited clinical relevance. Both issues can be mitigated by *in silico* modelling, *i.e*. mathematical descriptions of the biochemical processes solved on a computer, which provide a rapid assaying capability that can be used to guide subsequent experiments. *In silico* oral biofilm models are typically coarse-grained descriptions that do not permit the biofilm composition to evolve in time^17–20^. Recently, a two-dimensional cellular automata model based at the level of cell aggregates was developed to investigate competition in growing *S. gordonii-P. gingivalis* biofilms^21^ but clinical predictions for caries were not considered.

In the studies described here, computer simulations were employed to separate the independent effects of sugar frequency *versus* sugar amount on the cariogenicity of an *in silico* model for supragingival plaque. Both the frequency and total sugar intake were systematically varied over physiological ranges, and the terminal pH of the growing plaque biofilm quantified for each combination. Glucose was used as the model sugar due to the greater amount of published data on its metabolism by oral bacteria compared to sucrose, allowing for the relevant parameters from the literature to be employed with confidence. However, we expect no essential changes to our predictions if known parameters for sucrose were substituted, as long as the basic model assumptions held. This work extends preliminary studies using the same high-fidelity, aggregate-based model, developed by the authors to probe long-time changes in biofilm ecology, which investigated multiple factors influencing plaque composition and terminal pH^22^.

## Results and Discussion

The influx of nutrient (glucose) into the *in silico* model was measured in non-standard units that require clarification before results can be interpreted. Although dietary sugar intake is typically quoted in grams per day^13^, models such as ours require the concentration in saliva as an input. Converting grams to concentrations is possible with saliva flow modelling^17^, but requires additional parameters to be estimated, and introduces non-microbial phenomena, such as saturation at high intakes due to an increased salivary flow stimulated by the sugar. To focus solely on microbial determinants, flow was therefore neglected and the concentration of glucose input per day controlled, with units of g/L/d. For orientation, a daily intake of 15 g/L/d taken in four 15 minute pulses at 6 hour intervals corresponds to a concentration of 15 g/L during each pulse, and zero at all other times. This equates to a peak value of approximately 50 g/L in models with exponentially-decaying concentrations^17,19^, and is comparable to excess sugar growth conditions *in vitro* ^23–25^.

### Reversal of selection

The *in silico* system consisted of two populations of cell types that differed in their aciduricity, *i.e*. their ability to maintain high growth rates and metabolise sugars in low pH environments. The two populations were labelled NA, for non-aciduric cells with an optimum pH for glycolysis of 7.0, and A for aciduric cells that exhibit maximum metabolic rates at pH = 5; see Methods for details. The protocol adopted for this *in silico* system placed the populations of A and NA in competition for two resources, namely the nutrient supply and physical space. The nutrient diffuses across the saliva domain to the plaque where it is reduced by metabolic reactions, resulting in lower availability for particles deeper within the biofilm. In addition, the total particle count fluctuates around a constant value in which growth and division is balanced by acid-controlled death and the removal of particles farther than a distance *h*_*plaque*_ from the enamel, where *h*_*plaque*_ is a model parameter. This constancy of total particle number means proliferation of one lineage comes at the expense of another, resulting in a second source of competition.

**Figure 1:**
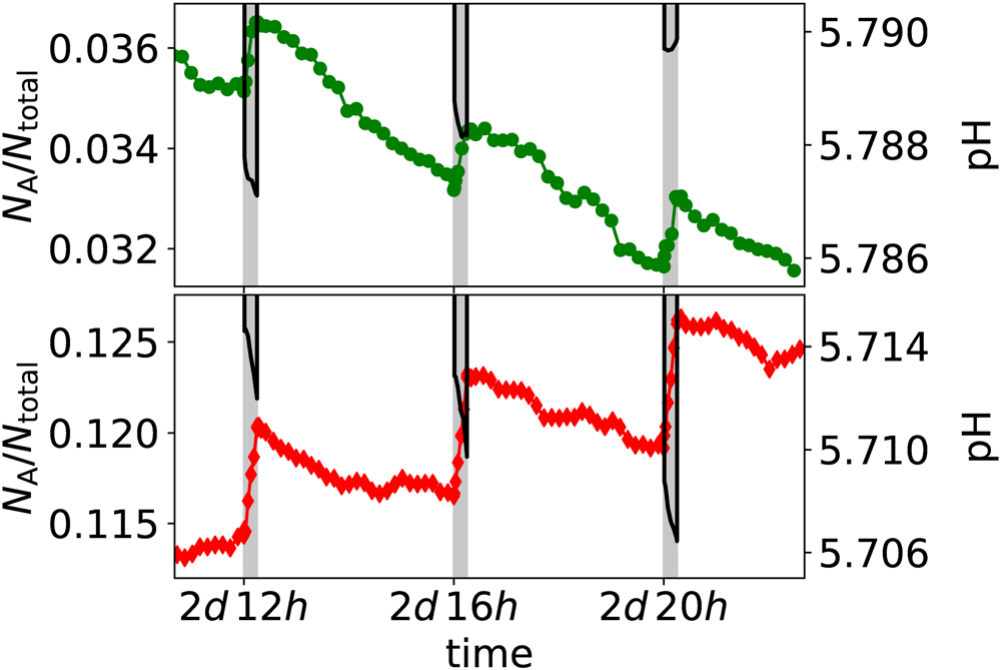
Examples of the coupled changes in biofilm composition and pH over a 12 hour period, with one 15 minute glucose pulse every 4 hours as highlighted by the vertical grey bars. The composition is expressed by the ratio of ${N}_{A}$, the number of aciduric (A) particles, to the total number of either type ${N}_{total}$. Note that the small variations in pH translate to much larger changes over periods of days. The top figure corresponds to a total sugar intake of 10 g/L/d, and the bottom figure to a system with twice that amount (20 g/L/d). In both figures, the symbols denote the fraction of particles that are of type A, with the values given on the left hand axes. Conversely the solid black lines show the pH measured at the enamel surface during each pulse, with values given on the right hand axes. Only the pH during a pulse is shown; the lowest pH between pulses is approximately 6.0 in both cases and is not plotted for clarity.

The glucose intake schedule imposed a feast-famine cycle with periods of high glucose availability during the pulse, interspersed with longer inter-pulse periods during which nutrient availability is limited to the low concentration of stored polyglucose [polyGl]. This resulted in a reversal in the direction of the competition at the start and end of each pulse; that is, A outcompeted NA during pulses, but NA outcompeted A in the inter-pulse periods, due to the very different environmental pH values between these two phases. Consequently, the fraction of the number of particles of type A relative to the total number of either type, *N*_*A*_/*N*_*total*_ with ${N}_{total}={N}_{A}+{N}_{NA}$, increased during pulses and decreased at other times. The variation in pH was contrary, decreasing when ${N}_{A}/{N}_{total}$ increased and *vice versa*. The net trend averaged over multiple pulses depended on the sugar intake schedule, with both high frequencies and high concentrations acting to increase *N*_*A*_/*N*_*total*_ and lower the pH. Examples for two different total carbohydrate amounts but the same frequency are given in Fig. 1. Note that although the pH changes between sequential pulses were small, more physiologically-relevant changes emerged over longer times as now discussed.

### Symbiotic to dysbiotic transition

**Figure 2:**
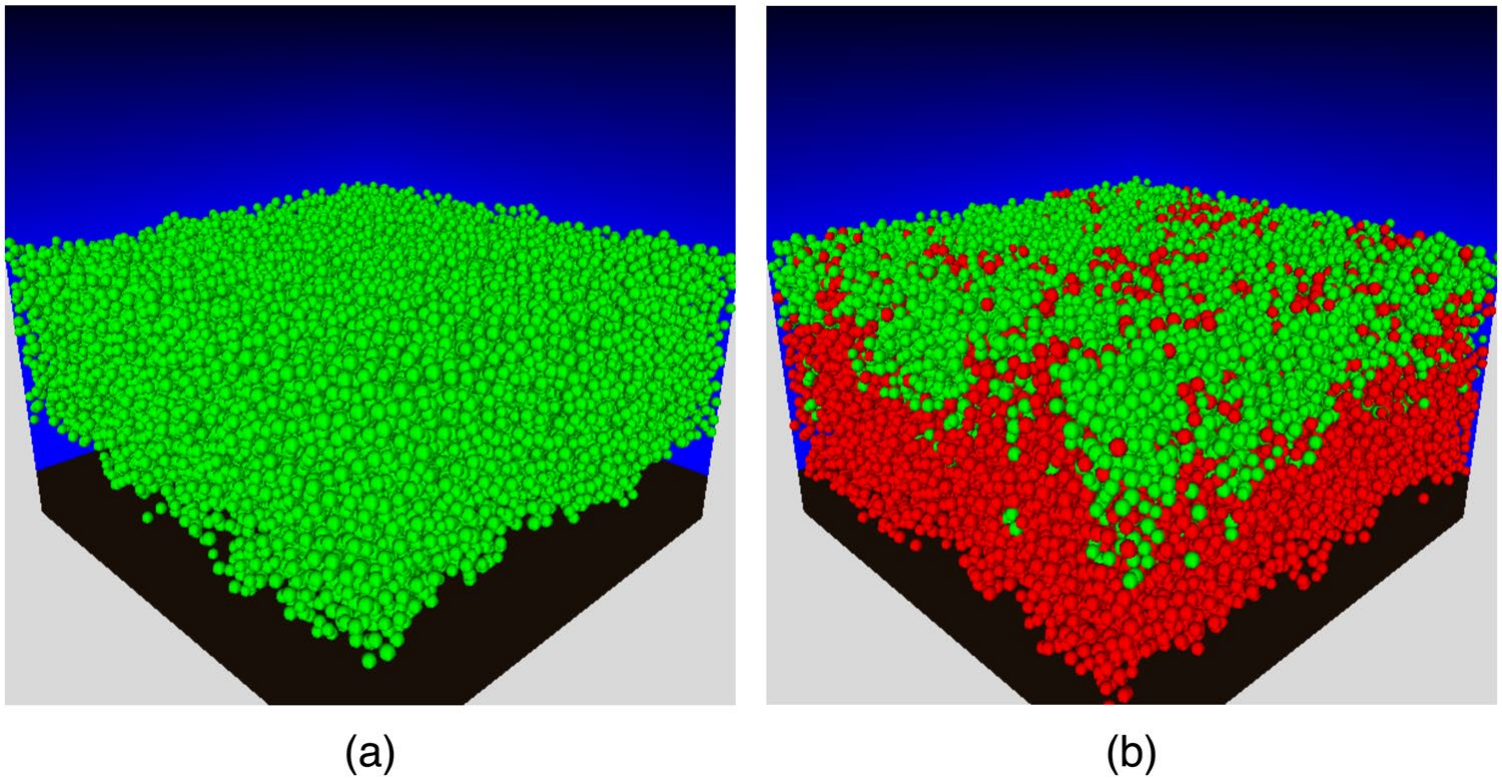
Snapshots of biofilm composition at t = 60d for a frequency of 6 glucose pulses per day, and a total daily amount of (**a**) 10 g/L/d and (**b**) 20 g/L/d. Green spheres represent particles of a non-aciduric cell type (NA), and red spheres represent those of an aciduric type (A). The brightness in the back planes is proportional to the concentration of acid. The computer simulations were initiated with 5% particles of type A and the remaining of type NA. Videos corresponding to snapshots (**a**) and (**b**) are available as Supplementary Videos S1 and S2, respectively.

A low sugar intake should lead to a symbiotic plaque biofilm that is dominated by non-aciduric, commensal oral bacteria that slow their conversion of sugar to acid as the environmental pH lowers, and are consequently incapable of driving the pH sufficiently low and sufficiently rapidly so as to initiate enamel demineralisation. Such cells are represented by the NA-type here. A snapshot of a model biofilm for a total intake of 10 g/L/d is given in Fig. 2(a), with the corresponding Supplementary Video S1. Dominance of NA after 60 days simulated pulsing is readily apparent.

The terminal pH reached during a sugar pulse for this example never drops below approximately 5.79, insufficient for enamel demineralisation; hence cariogenicity is low and the biofilm remains in a symbiotic relationship with the host. By contrast, doubling the total sugar intake to 20 g/L/d, with all other parameters fixed, leads to a dysbiotic biofilm in which particles of type A outnumber those of type NA over time, as shown in Fig. 2(b) and Supplementary Video S2. The terminal pH now drops to approximately 4.92, lower than the 5.5 required for net enamel demineralisation, corresponding to high cariogenicity and the development of a dysbiotic plaque. Thus, varying the sugar intake in the model drives plaque composition and cariogenicity in broadly the expected manner.

### Net selection and critical intake frequency

**Figure 3:**
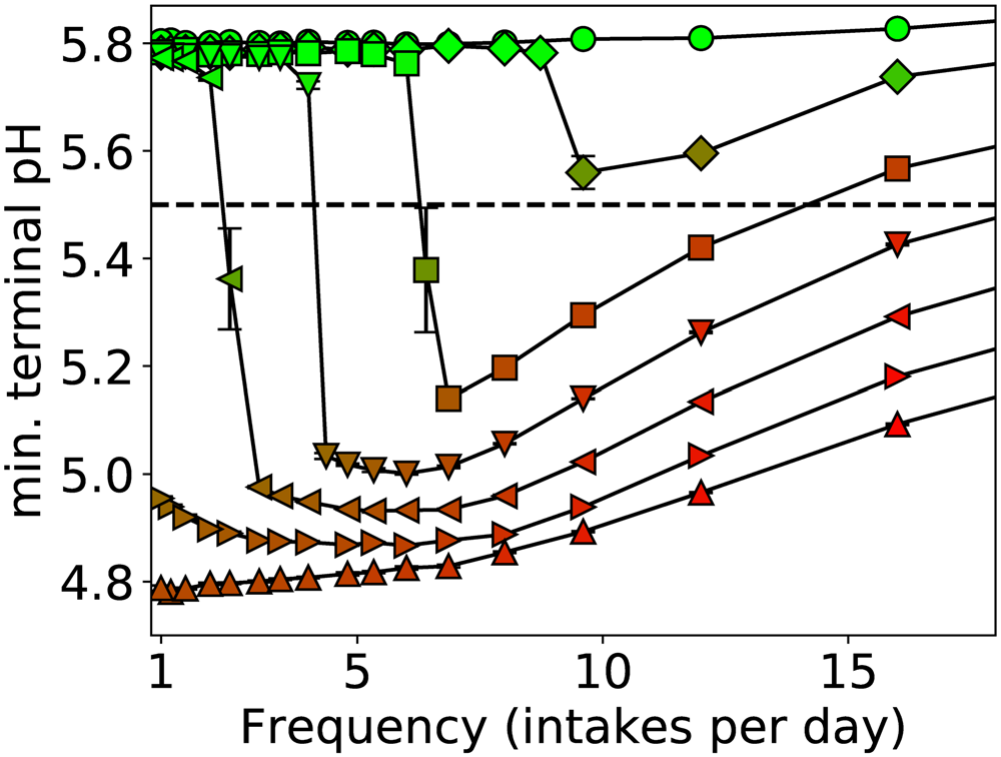
Minimum pH *versus* frequency of intake taken at a time of 60 days, where each line corresponds to a different total amount of glucose as indicated in the legend. From top to bottom, the curves correspond to total amounts of 10 g/L/d (○), 12.5 g/L/d (◊), 15 g/L/d (□), 17.5 g/L/d (▽), 20 g/L/d (◁), 22.5 g/L/d (▷), and 25 g/L/d (△). Markers are coloured according to ${N}_{A}/{N}_{total}$ with *N*_*A*_ the number of aciduric (A) particles and *N*_*total*_ the total number of particles of either type, with red for high values and green for low values, and the line pH = 5.5 below which enamel dissolution is expected to occur has been highlighted. Sample size n = 5 per point and standard errors shown.

The earliest presentation of caries is the occurrence of white spots on the tooth surface, resulting from high environmental acidity that drives demineralisation^7^. We used the minimum pH at the enamel surface, measured over a time interval including at least one glucose pulse, as our metric of plaque cariogenicity, in the absence of explicit enamel de- and remineralisation reactions^19,20^. The pH after t = 60d of glucose pulsing is plotted in Fig. 3 for a range of physiologically-relevant total daily amounts and frequencies. The biofilm composition in terms of *N*_*A*_/*N*_*total*_ is also shown. For a given frequency, increasing the total amount both lowered the minimum pH and increased the fraction of A in the biofilm. This is to be expected, as the greater sugar availability is metabolised to higher concentrations of acid, which selects particles of cell type A over those of type NA.

The critical pH = 5.5 for the onset of demineralisation is also shown in Fig. 3 to gauge clinical impact; data points below this line correspond to potentially cariogenic biofilms. Three classes of biofilm cariogenicity were revealed. For low total amounts of 12.5 g/L/d of glucose or less, the pH remained above 5.5 at all times, and therefore the biofilm never became cariogenic, irrespective of the frequency of sugar intake. Conversely, for high total amounts of 22.5 g/L/d or greater, the biofilm was always cariogenic, again irrespective of frequency. It was only for intermediate total amounts that frequency was a determinant between cariogenic and non-cariogenic plaque, as is evident in the figure, where only the curves for intermediate totals exhibit a transition from pH > 5.5 to pH < 5.5. The frequency at which this occurs depended on the total intake, with an increasing amount of glucose broadening, to include lower frequencies, the range of occurrences of intake that could be classified as being potentially cariogenic. The model parameters are based on *in vitro* experiments as stated in the Table 1, therefore, this finding should be regarded as one of potential clinical relevance.

**Table 1:** Summary of key model parameters. Values refer to those used unless otherwise stated.

Label	Parameter	Value	Based on
*h*_*plaque*_	Biofilm thickness	250 μm	41
*h*_*saliva*_	Thickness of the saliva layer	250 μm	Assumed
*X*,*Y*	System box width, breadth	500 μm	Assumed
${K}_{A}^{Gl}$	Glucose half-saturation for A	0.4 g/L	19
${K}_{NA}^{Gl}$	Glucose half-saturation for NA	0.1 g/L	19
${K}_{A}^{acid}$	Optimal [H^+^] for A	10^−5^ mol	19
${K}_{NA}^{acid}$	Optimal [H^+^] for NA	10^−7^ mol	Estimated based on^25^
${\mu }_{max}$	Glycolysis base rate (A, NA)	5/h	42
*f*	Fraction of total for [polyGl]	1%	Estimated based on^20^
*Y*	Growth factor	0.1	42
*Y* ^*EPS*^	EPS production factor	0.04	Assumed
${r}_{A}^{death}$	Death rate for A at neutral pH	3 × 10^−3^/h	43
${r}_{NA}^{death}$	Death rate for NA at neutral pH	6 × 10^−3^/h	43

**Figure 4:**
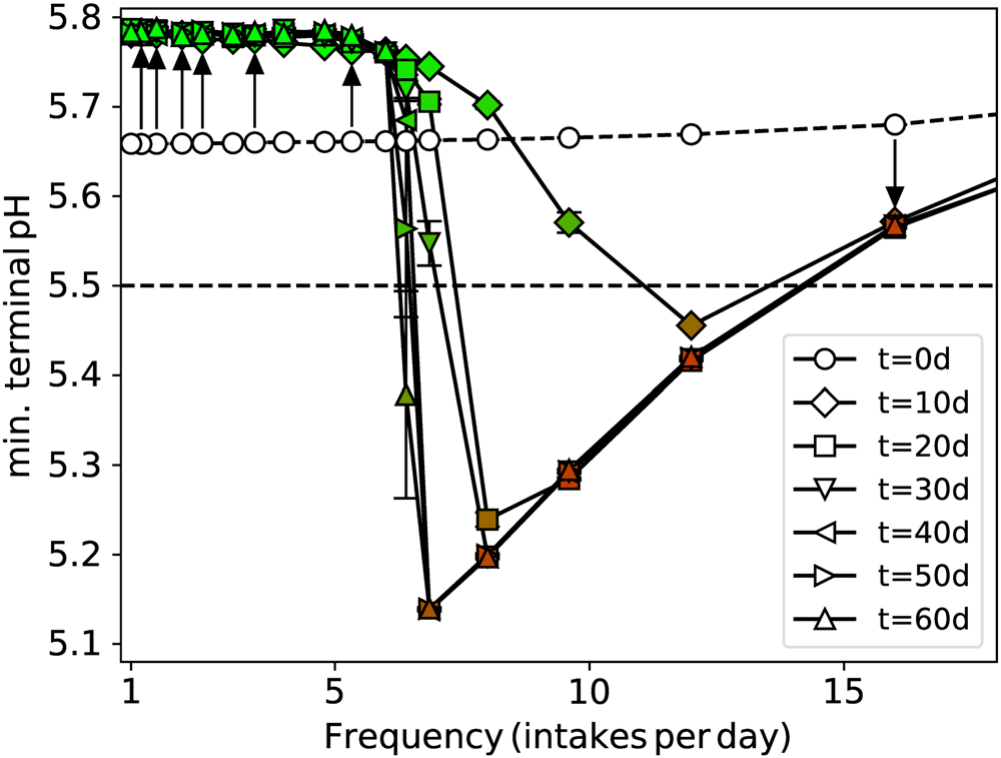
Variation of the minimum pH reached during a glucose pulse with time for different intake frequencies but the same total amount 15 g/L/d. Data are shown at 10 day intervals as indicated by the legend. The arrows point in the direction of increasing time for selected points, to help guide the eye. Symbols are coloured according to biofilm composition, ${N}_{A}/{N}_{total}$, so green means NA-dominated (non-aciduric) and red means aciduric (A)-dominated. The critical pH = 5.5 for enamel demineralisation is also shown. Sample size n = 5 per point and standard errors shown.

The time evolution of the symbiotic to dysbiotic transition is demonstrated in Fig. 4, where the minimum pH and biofilm composition is shown over 10d intervals for a single total amount. It is evident that for infrequent pulsing with a corresponding high glucose concentration, the biofilm remained dominated by particle type NA, and the pH never dropped below 5.5. In this regime, the sugar concentration far exceeded the saturation threshold imposed by microbial metabolism, and most of the available nutrient was not converted to acid. This corresponds to the established view that frequency is the sole determinant of plaque cariogenicity. Very frequent intakes with low concentrations also resulted in a commensal biofilm with both low acid production and caries potential, despite the drift in biofilm composition to include a substantial fraction of particles of type A.

Lying in between these two extremes was the critical frequency at which the fate of the biofilm abruptly changed from NA-dominated with only a modest pH change, to A-dominated with a pH substantially below the 5.5 necessary to initiate net enamel dissolution. Dominance of A or NA was quickly achieved far from this critical frequency, but many days of pulsing were required to observe the eventual fate of the biofilm near to the critical value. A similar phenomenon was observed in our earlier study in which all pulses had super-saturation concentration, and the critical frequency was found to depend on a range of model parameters^22^.

### Adaptation to acidic environments

**Figure 5:**
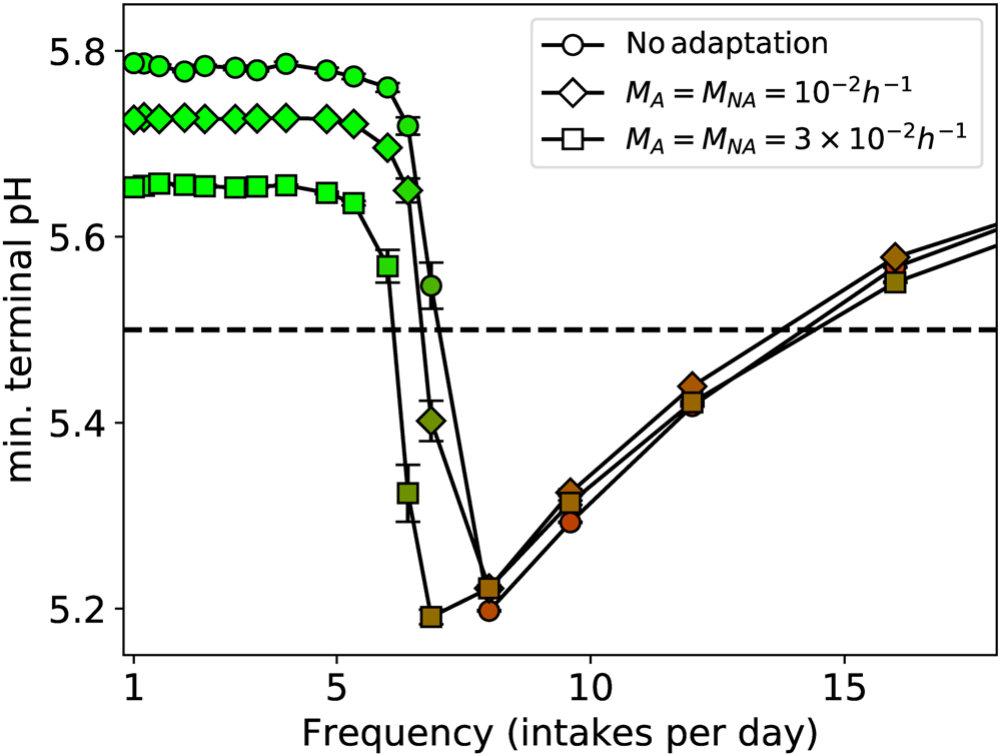
Minimum pH *versus* frequency of sugar intake for two rates of adaptation, with the data for no adaptation also shown for comparison. The total amount of sugar is the same for all cases (15 g/L/d), and the data were taken after 60 days of growth. Markers are coloured to ${N}_{A}/{N}_{total}$, with red for high values (*A* dominance) and green for low values (*NA* dominance). Sample size n = 5 per point and standard errors are shown. A = aciduric; NA = non-aciduric.

It has been suggested that a precursor of the transition to dysbiotic plaque is the adaptation of commensal bacteria to the environmental acidity, increasing the net acid production, and hence favouring the succession of aciduric species^11,26^. Being a dynamic process, the degree of adaptation will depend on the durations of high and low-pH episodes, *i.e*. the sugar pulsing schedule. Adaptation was modelled as the continuous change of each particle’s optimum pH for glycolysis, towards the current environmental pH value, at species-dependent rates ${M}_{A,NA}$ calibrated against *in vitro* experiments (see Methods for details). These changes were assumed to be non-inheritable. The terminal pH for a single total intake for two rates of adaptation are plotted in Fig. 5, alongside the no-adaptation control. Two consequences are apparent. Firstly, there was a lowering of the pH in symbiotic plaque developed during infrequent sugar pulses, which nonetheless remains above the threshold for enamel demineralisation. Secondly, with increasing rates of adaptation the lower frequency at which the biofilm becomes dysbiotic was slightly reduced, *i.e*. from 7 pulses per day without adaptation to a little over 6 pulses per day for the higher adaptation rate.

These qualitative observations are in agreement with the hypothesis of Takahashi^11,26^. However, the magnitudes of the changes were small, despite the larger of the two rates most likely being an over-estimate. Interrogation of the raw simulation variables revealed an explanation. The doubling time for a newly-formed particle to grow and divide was consistently around 10 hours, comparable to the slower growth rates predicted for mature nutrient-depleted biofilms^27,28^. Over such times, and with the higher adaptation rate, the optimum [*H* ^+^] changes by around 10% for A, and 60–70% for NA, both of which are small when converted to the pH scale. Hence the quantitative effect is minimal. We propose that adaptation is responsible for potentially measurable but weak changes to ecological shifts in supra-gingival plaque, although it should be noted that exact calibration with the planktonic experiments was imperfect and thus these predictions should be regarded as tentative.

### Spatial gradients

**Figure 6:**
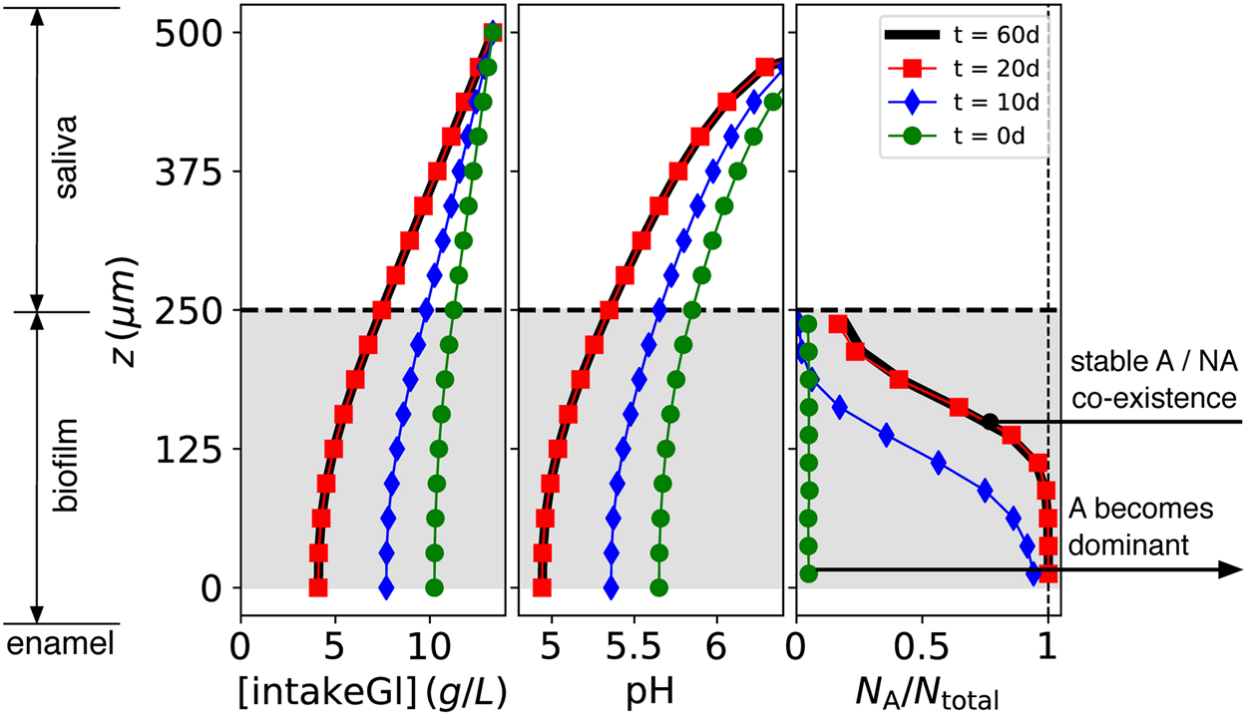
Spatial variation of the concentration of glucose [intakeGl], pH, and biofilm composition ${N}_{A}/{N}_{total}$, as a function of depth *z* for the times shown in the legend. Note that the thick black line for t = 60d almost perfectly coincides with the data points for t = 20d. All data are taken during a glucose pulse. The horizontal dashed line denotes the free surface of the biofilm at *h*_*plaque*_ = 250 *μm*, and the saliva-air interface is at ${h}_{plaque}+{h}_{saliva}=500\mu m$. The region occupied by biomass is shaded light grey. The total intake was 20 g/L/d, with a pulse every 4 hours. Other parameters are as in Table 1. Standard errors were smaller than the symbols and not shown for clarity. A = aciduric; NA = non-aciduric.

*In vivo* studies have revealed that plaque acidity is not uniform in carious cavities, but decreases with distance from the enamel surface^29^. This could be due to purely physicochemical processes, but may also reflect a spatially heterogeneous population of aciduric bacteria. To probe this possibility, plotted in Fig. 6 is the variation of nutrient concentration [intakeGl], pH, and *N*_*A*_/*N*_*total*_, with distance from the enamel, all measured during a pulse. It is clear that the fraction of A particles was indeed greater near the enamel surface, reflecting the greater acidity in this region as also evident from the figure. In addition, the concentration of nutrient smoothly decreased to a finite value as the enamel surface was approached. This mass transfer limitation was due to nutrient uptake in the upper biofilm region depleting the concentration as the distance from the site of introduction increased^30,31^. It is not due to reduced diffusion within the biofilm, as the diffusion coefficient *D* was uniform.

The biofilm composition slowly changed with time, and this had a demonstrable effect on the nutrient and acid gradients. For the parameters of Fig. 6 the initial increase in numbers of particles of type A was near the enamel surface, where the pH was the lowest and the selective advantage greatest, and this in turn led to an increase in nutrient uptake and acid production. These conditions further enhanced the growth of A particles relative to NA, and the A-rich region expanded farther from the enamel with time. If unchecked, this positive feedback loop would result in complete dominance of A throughout the film. However, with both high and low pH inhibiting growth as in equation (1), particles of NA near the saliva interface enjoyed a competitive advantage over A during the inter-pulse periods, when glucose availability was sparse and pH exceeded 6, resulting in the stable coexistence of both types.

## Conclusion

The established view that plaque cariogenicity depends on the frequency of intake of dietary sugars rather than on the total daily amount ingested was recently challenged by large-scale longitudinal studies, which concluded that clinical presentations of caries were more strongly correlated with the total intake^13^. By employing a computational model of supra-gingival plaque that is capable of sampling more combinations of frequency and total amount than could easily be assayed *in vitro*, we have concluded that the total intake of dietary sugars is a determining factor, but in a manner that can also depend on the frequency. For very low or very high total amounts of dietary sugar intake, the plaque is never or always cariogenic, respectively, independent of the frequency. Frequency is a critical factor for intermediate totals of sugar intake, including the estimated average consumption^17,19,23–25^, with high frequencies leading to a terminal pH that is predicted to drive enamel demineralisation and initiate caries.

The model presented here could support public guidance for dietary regimens leading to improved oral healthcare. Attempts to translate these findings to such public guidance should be tempered by the realisation that fixing one parameter (either sugar frequency or total amount) and systematically varying the other, while useful for extracting their independent contributions, is not how people typically schedule their diets. Advice on altering dietary regimens to reduce the incidence of caries should always be presented in the context of the combined effect of both factors. The theoretical nature of the methodology, however, has limitations that are common to *in silico* biofilm modeling, namely the various simplifying assumptions required to close the equations, employing parameters from planktonic *in vitro* experiments, and taking cell aggregates rather than individual cells as the basic units to reduce the computational load. However, our model generated realistic predictions for the spatial gradients of carbohydrate quantities of interest, and was parameterised by historical experimental data, so our predictions should be regarded as physiologically plausible.

## Methods

Our computational model belongs to the family of individual or agent-based schemes that have been extensively employed in studies of environmental and industrial biofilms^32,33^. The bacterial population was divided into two groups defined by their function: aciduric cells, denoted A, which are capable of metabolising sugars to acid in a low pH environment, and non-aciduric cells labelled NA for which such conversion drastically slows as the pH is lowered. Data to define the properties of these two groups in the modelling studies were taken from previously published work on bacteria that are linked to enamel health or have been implicated with caries (see Table 1). All cell types were assumed to be acidogenic, i.e. capable of metabolising glucose to lactic acid. The model is described below, with an emphasis on those features that differ from our previous work^22,34–36^. Snapshots are given in Fig. 2 for two different total amounts of glucose, with corresponding Supplementary Videos S1 and S2.

### System geometry

**Figure 7:**
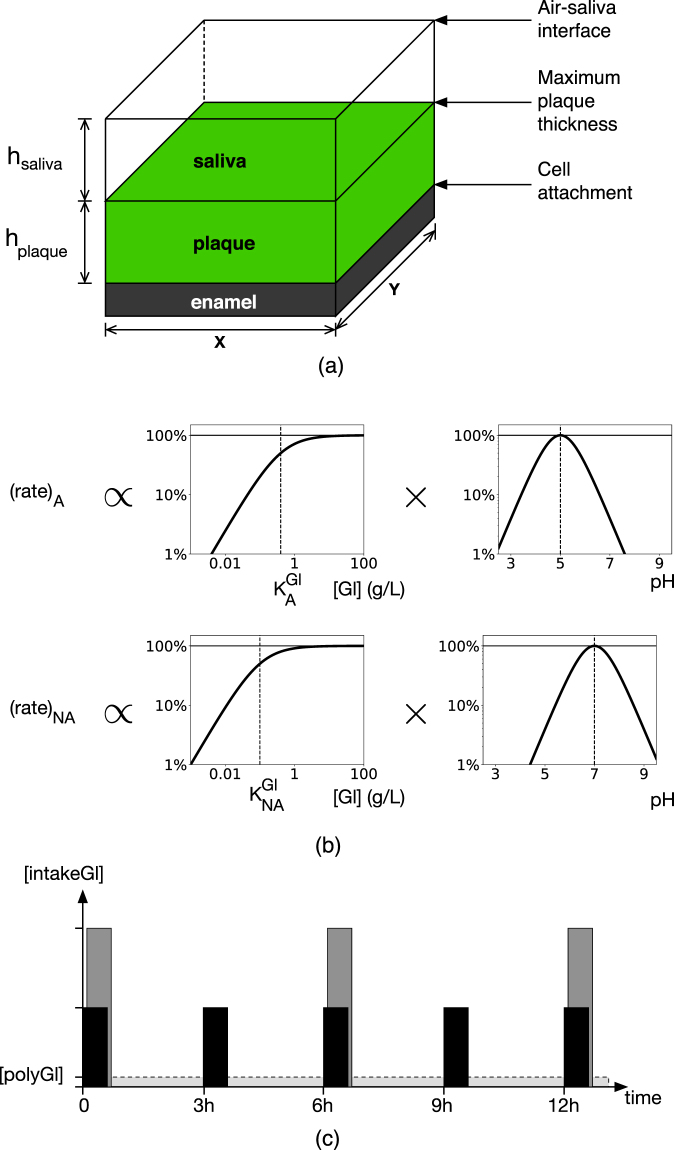
Schematic representation of key model components. (**a**) The model domain included a plaque biofilm region adjacent to the enamel surface, and a more distant saliva region. Periodicity was assumed along the x and y-axes. (**b**) Graphical representation of the glycolytic rates for cell types A and NA, with a saturation factor for glucose availability ([Gl] of either [intakeGl] or [polyGl]), and an optimum pH of 5 and 7 for A and NA, respectively. Cellular growth was proportional to the glycolytic rates. (**c**) Schematic representation of periodic sugar intake showing two different frequencies, one pulse every 3 hours (black) and once every 6 hours (grey), with the same total amount. The constant, low concentration of [polyGl] is also shown. A = aciduric; NA = non-aciduric.

The plaque biofilm was embedded in a rectangular domain representing a region of saliva adjacent to the enamel surface; see Fig. 7(a). Particles corresponding to cell aggregates are attached to the substratum, and grow and divide according to the metabolic rules described below. All particles exceeding a pre-defined height ${h}_{plaque}$ were removed from the system after each growth phase. The saliva layer was of a predetermined thickness ${h}_{saliva}$, so the saliva-air interface lay at a total distance ${h}_{plaque}+{h}_{saliva}$ from the enamel. As our focus is on stagnant sites we considered values of ${h}_{saliva}=\,$250 μm in excess of the mean thickness 100 μm^37^.

Permeating both domains were scalar fields corresponding to the concentrations of three dispersed phases: the dietary sugar intake [intakeGl], which is glucose in this model because of the greater amount of published data on the bacterial metabolism of this carbohydrate, the stored polyglucose [polyGl], and the concentration of lactic acid [acid]. Acid dissociation and pH followed empirical data^38^. The polyglucose stored during sugar-rich phases was represented as a low-level nutrient source that was utilised at all times. This was modelled as a constant and uniform concentration that was 1% of the total sugar intake under consideration. The scalar fields are summarised in Supplementary Table S1.

### Glycolysis and metabolism

The conversion of glucose and stored polyglucose to lactic acid was modelled by the rate equation (1), with different parameters for the particle type A or NA. If particle (bacterial aggregate) $i$ had mass ${m}_{i}$ and was of type A, its glycolysis rate ${r}_{i}$ was

1 $${r}_{i}={m}_{i}{\mu }_{max}\frac{[Gl]}{[Gl]+{K}_{A}^{Gl}}\frac{4[{H}^{+}]{K}_{A}^{acid}}{{([{H}^{+}]+{K}_{A}^{acid})}^{2}},$$

with a similar expression for NA. (1) is represented diagrammatically in Fig. 7(b). ${\mu }_{max}^{A}$ is the base rate, $[Gl]$ is the concentration of either dietary glucose or stored polyglucose, and $[{H}^{+}]$ is the concentration of *H* ^+^ ions after acid dissociation. The factor for acid inhibition reduces metabolism either side of an optimal pH at $-lo{g}_{10}({K}_{A}^{acid})$. The parameters were based on *in vitro* studies of bacterial strains representative of pathogenic and commensal plaque, see the Table 1. The rates for [intakeGl] and [polyGl] were calculated separately and added to give the total acid production.

Growth was tightly linked to metabolism: the rate of mass increase was a constant factor $Y$ times the rate of glycolysis, $\frac{d{m}_{i}}{dt}=Y{r}_{i}$, and another constant factor *Y* ^*EPS*^ for the rate of EPS production. Note that while glucose and sucrose are expected to generate differing quantities of EPS, corresponding to different values of *Y* ^*EPS*^, the parameter sensitivity heat map of our previous study^22^ showed only small quantitative changes to plaque composition and terminal pH on changing *Y* ^*EPS*^, as compared to other model parameters such as *e.g*. ${K}_{A}^{acid}$ (Fig. 7 of ^22^). Particles divided when they reached a maximum mass, when they were replaced by two daughter particles of the same total mass. Particle death due to environmental pH was included, with higher rates for more acidic environments,

2 $${r}_{i}^{death}={r}_{A}^{death}\frac{[{H}^{+}]}{{10}^{-7}},$$

where the particle-dependent constants ${r}_{A}^{death}$ and ${r}_{NA}^{death}$ obey ${r}_{A}^{death} < {r}_{NA}^{death}$ to reflect the greater acid tolerance of A. After death, the particles were removed from the system – there was no utilisation of residual biomass. After each phase of growth and death, particles repositioned to satisfy the mechanical equilibrium of the entire biofilm in what was a straightforward extension of the two-dimensional method^22^.

### Adaptation

Adaption is here defined as the ability of an organism to modify its physiology within a generation in response to external changes, so as to increase its net growth and hence survival probability. More specifically, we considered adaptation to local acidity [*H* ^+^], which was modelled by moving each particle’s ${K}_{A,NA}^{acid}$ towards [*H* ^+^] at a species-dependent rate ${M}_{A,NA}$, *i.e*.

3 $$\frac{d{K}_{A,NA}^{acid}}{dt}={M}_{A,NA}([{H}^{+}]-{K}_{A,NA}^{acid}),$$

so that the optimum acidity for glycolysis moves towards the current environmental acidity. Such changes are not inherited, *i.e*. on division, both daughter particles revert to the initial values for their cell type. Note that the ${M}_{A,NA}$ refer to absolute changes in ${K}_{A,NA}^{acid}$; the relative changes will depend on both ${M}_{A,NA}$ and the initial values of ${K}_{A,NA}^{acid}$ given in the Table 1.

${M}_{A,NA}$ were calibrated against *in vitro* experiments^39^, identifying NA with *S. sanguinis* (formerly *S. sanguis*) and A with *S. mutans*. The protocol was to drop pH from pH = 7 to a controlled lower value for a specified period of time, and measure the rate of acid production just after pH = 7 was restored. To improve correspondence with the experiments, where cell viability remained high throughout, cell death was set to zero for this calibration. Although exact calibration was not feasible using the simplified expression for glycolysis in equation (1), broad agreement was achieved for ${M}_{A}={M}_{NA}={10}^{-2}{h}^{-1}$ and ${M}_{A}={M}_{NA}=3\times {10}^{-2}{h}^{-1}$, as shown in Supplementary Fig. S1 (to be compared with Fig. 1 of ref.^39^).

### Simulation protocol

All particles were initially placed in an ordered configuration up to a height ${h}_{plaque}$, with 5% of type A and 95% of type NA uniformly distributed throughout the biofilm. Glucose was introduced by periodically setting the value of [intakeGl] at the saliva-air interface to be a non-zero value such that the total intake was fixed, so doubling the frequency halves the magnitude as shown in Fig. 7(c). Each pulse lasted a fixed period of 15 minutes to approximate the typical duration of low-pH after a sugar pulse^38^. Varying the details of the saliva-plaque exchange mechanism is not expected to significantly alter our core findings^40^. Values of [intakeGl] should be multiplied by 3.0 when comparing to peak values of models with exponentially-decaying concentrations (*e.g*.^19^) to give the same net amount.

## Electronic supplementary material

Supplementary information

Supplementary Video S1

Supplementary Video S2
