# Supplementary material for: In silico modelling to differentiate the contribution of sugar frequency versus total amount in driving biofilm dysbiosis in dental caries
Source: Sci Rep. 2017 Dec 12;7:17413. doi: 10.1038/s41598-017-17660-z (PMC5727185; doi:10.1038/s41598-017-17660-z)
Supplement: Supplementary file 1 — Supplementary information [file 41598_2017_17660_MOESM1_ESM.pdf]

In silico modelling to differentiate the contribution of sugar frequency _versus_ total amount in driving biofilm dysbiosis in dental caries

David Head, Deirdre Devine, P D Marsh

<u>Contents</u>

Page 2: Supplementary Table S1: Summary of the dispersed phases in the model.

Page 3: Supplementary Figure S1: Calibration assays for the adaptation parameters in the model.

Page 4: Supplementary Videos S1 and S2: Videos for the same parameters as Fig. 1(a) and Fig. 1(b) in the main text. Video files provided separately.

Label	Meaning	Spatial varying?	Boundary conditions
[intakeGl]	Glucose intake	Yes	Given value at air-saliva interface.<br>No flux through enamel surface.
[acid]	Lactic acid	Yes	Zero at air-saliva interface.<br>No flux through enamel surface.
[polyGl]	Polyglucose storage	No	n/a

Supplementary Table S1: The dispersed phases included in the model, with boundary conditions for those that vary spatially.

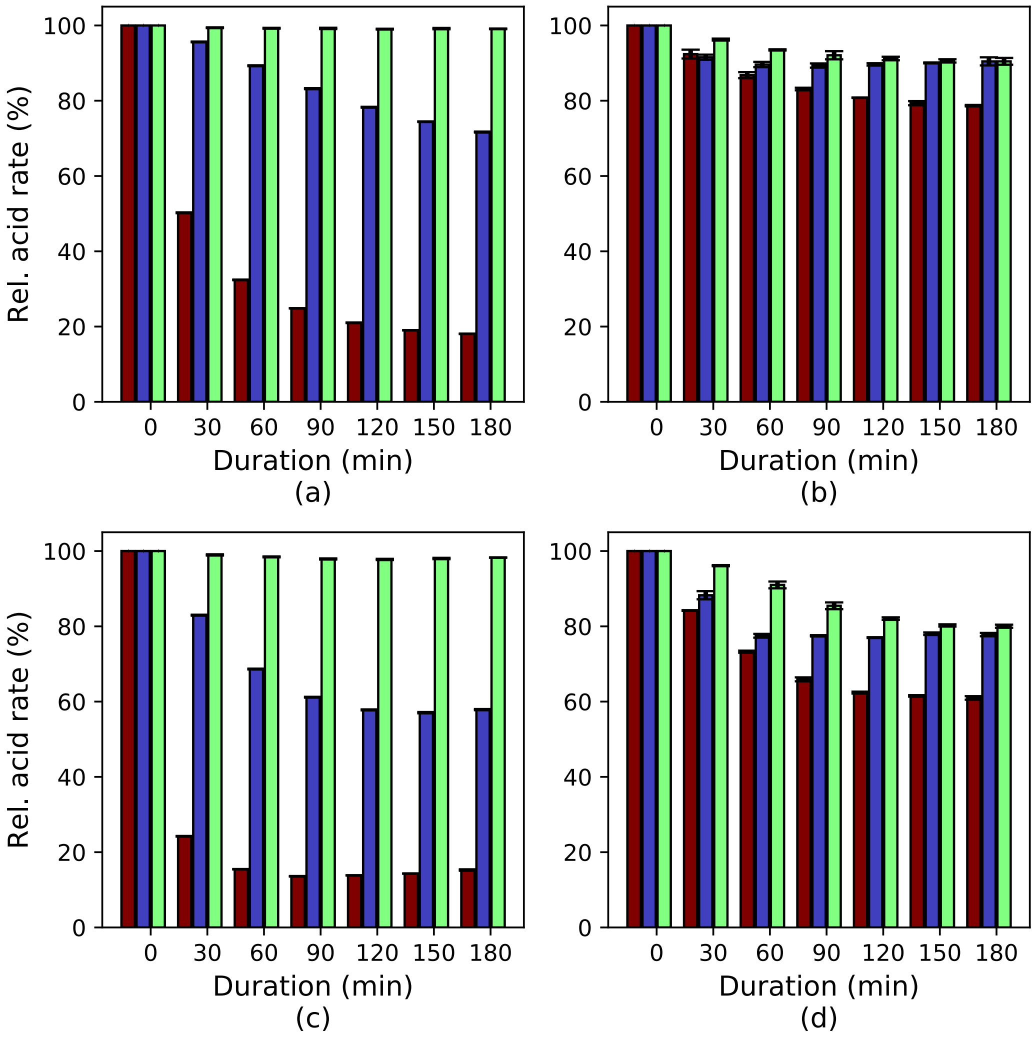

Supplementary Figure S1: The relative drop in acid production rate on recovery of pH=7 after the pH was lowered to one of pH=4 (dark red bars), pH=5 (blue bars) or pH=6 (light green bars) for the specified duration, for mono-species systems of particles of type NA in the left panes (a,c), or A on the right panes (b,d). The adaptation rates were $M_A = M_{NA} = 10^{-2}h^{-1}$ for the top panes (a,b) and $M_A = M_{NA} = 3\times10^{-2}h^{-1}$ for the bottom panes (c,d).

Supplementary Video S1: 60 days biofilm evolution for an intake of 10 g/L/d and 6 glucose pulses per day, the same parameters as Fig. 1(a). Green spheres denote particles of type NA, and red spheres those of type A. The blue background is calibrated to the concentration of acid, with the maximum shown in the upper bar (note that this is the concentration prior to the disassociation calculation).

Supplementary Video S2: Same as Supplementary Video 1, but with double the total amount of glucose intake, *i.e.* 20 g/L/d, the same parameters as Fig. 1(b).
